# Supplementary material for: Are carbon emissions trading and green financial instruments synergistic? -Comprehensive quantitative research based on content analysis
Source: PLoS One. 2024 Mar 7;19(3):e0298601. doi: 10.1371/journal.pone.0298601 (PMC10919601; doi:10.1371/journal.pone.0298601)
Supplement: S1 Data — (ZIP) [file pone.0298601.s001.zip › Data/地级市Z_F工作报告.zip!/╦─┤¿/│╔╢╝╩╨/└┤╫╘╣1⁄2╓┌║┼í╛┬φ┐╦╩2╛▌═°í┐_2019.docx]

各位代表：

现在，我代表市人民政府，向大会报告工作，请予审议，并请市政协委员提出意见。

一、2018年工作回顾

2018年是成都发展史上极不平凡的一年。习近平总书记亲临四川视察，嘱托成都加快建设全面体现新发展理念的城市，为新时代治蓉兴蓉指明了前进方向、提供了根本遵循。省委十一届三次、四次全会首次把成都定位为全省“主干”，支持成都跨越龙泉山向东发展，为成都发展开创了历史机遇。市委十三届三次、四次全会为实现新时代成都“三步走”战略目标明晰了实践路径。

一年来，我们以习近平新时代中国特色社会主义思想为指导，深入学习贯彻党的十九大和十九届二中、三中全会精神，在党中央国务院、省委省政府和市委坚强领导下，在市人大、市政协的监督支持下，紧紧依靠全市人民，推动全市高质量发展取得积极进展。全年各项经济指标全面完成，预计地区生产总值超过1.5万亿元、增长8%，全社会固定资产投资增长10%，社会消费品零售总额增长10%，一般公共预算收入同口径增长9.4%，城乡居民人均可支配收入分别增长8.2%、9%。切实履行政府职能，按照“全面落实年”部署，重点开展了以下工作：

（一）推动经济高质量发展。加快产业功能区建设。实施高质量现代化产业攻坚计划，加快构建“5+5+1”现代产业体系，制定66个产业功能区总体规划，编制“两图一表”，新开工建设标准厂房510万平方米、园区配套住房907万平方米。不断提升制造业能级。电子信息、装备制造、医药健康、新型材料、绿色食品五大产业增加值增长10.8%，占全市工业增加值比重达82%。电子信息产业支撑有力，新型显示、软件、信息安全等领域跻身全国前列。汽车产业稳步发展，整车产量达130万辆。轨道交通产业已具备地铁、有轨电车、悬挂式单轨等整车制造和维保能力。生物医药产业加快发展，投资、主营业务收入均增长20%以上。医美产业快速突破，新增医美机构131家、增长48.7%。氢能产业链初步形成。积极推进5G产业布局，建成全国首个5G示范街区，二环快速公交成功实现全球首例户外5G测试。项目支撑切实强化，签约引进京东方高世代AMOLED、安谋科技、比亚迪新能源汽车、国科量子通讯设备等先进制造业重大项目195个，药明康德生命健康产业园、紫光成都存储器制造基地等143个重大项目开工建设，中电熊猫成都8.6代液晶面板生产线等120个重大项目竣工投产。规模以上工业增加值增长8.5%。提升发展服务业。大力发展金融服务、现代物流、会展经济、文化旅游、生活服务五大新兴服务业，服务业增加值增长9%。加快建设西部金融中心，本外币存款余额3.8万亿元、贷款余额3.3万亿元，新增上市公司11家，深交所西部基地落户运营。高起点建设世界旅游名城，签约引进中旅集团等知名企业，大力开展主题营销，实现旅游总收入3712.6亿元，旅游总人数2.4亿人次，其中入境游客340.6万人次。高水平建设国际会展之都，成功申办第六十届泛美开发银行理事会年会，成功举办中国西部国际博览会、蓬皮杜“全球都市”国际艺术双年展等重大展会活动689个，会展总收入增长17.2%。高标准建设国际美食之都，推广特色街区餐饮标准化管理、精细化标示，餐饮收入增长13.7%。电子商务交易额达1.7万亿元、增长27%。加快发展都市现代农业。着力推进现代农业产业功能区（园区）建设，新建高标准农田43万亩，粮食安全得到有效保障。“三品一标”认证数达1347个。农商文旅体加快融合发展，农业增加值增长3.6%。推动民营经济持续健康发展。推进以创新和成长为导向的企业扶持激励制度改革，出台促进民营经济健康发展25条，建立100亿元上市公司纾困帮扶基金，开展集中清理拖欠民营企业账款专项行动。新增民营企业9.35万户。大力发展新经济培育新动能。启动实施新经济企业梯度培育计划和“双百工程”，开展共享停车、共享办公、社区消费等场景应用，大力推动人工智能、区块链、虚拟现实等新兴技术发展与应用，独角兽岛等空间载体加快建设。新增新经济企业4.1万户，培育独角兽企业4家，新经济活力居全国城市前列，成都正成为最适宜新经济成长的城市！

（二）依法加强市场监管。加强品质成都建设。推动九大领域标准升级，加快建设国家技术标准创新基地。中航工业成飞研究所、中电子二十九所潘玉华获第三届中国质量奖，市政府获首批中国质量奖政府提名奖。康弘药业获第五届“中国工业大奖”。成都华气厚普机电公司等16家单位获“市长质量奖”。创新市场监管模式。强化事中事后监管，深入推进“双随机、一公开”监管。加强市场主体信用监管，完善市公共信用信息系统。坚持包容审慎监管，对初创企业实行“包容期”管理和审慎性执法。加强重点领域监管。开展知识产权保护“雷霆”行动。新设21个版权服务工作站。强化“两类公司”监管，大力打击和处置非法集资，守住了不发生系统性区域性金融风险的底线。严格执行国家各项调控政策，房地产市场平稳健康发展。切实加强市场价格监管，居民消费价格上涨1.4%。强化食品药品安全监管，积极创建国家食品安全示范城市，持证餐饮单位85%以上实现明厨亮灶。开展疫苗、中药饮片等领域专项整治。加强产品质量安全监管，开展“质检利剑”、有机产品认证等专项整治行动，努力让市民买得放心、用得舒心、吃得安心！

（三）着力提升社会治理水平。加快建设和谐社区。实施高效能社会治理攻坚计划，加快推进城乡社区发展治理“五大行动”，建成社区综合体79个、示范性国际化社区4个，城乡社区发展治理新模式荣获全国“民生示范工程”第一名。深化平安成都建设。完善立体化信息化社会治安防控体系，推广“1+3+N”专群联动机制。深入开展扫黑除恶专项斗争，加快建设综治中心、“雪亮工程”和“群众工作之家”。开展“净网2018”专项行动，成功承办第五届国家网络安全宣传周活动。切实推动城市安全发展。积极创建国家安全发展示范城市，不断提升应急管理能力，规划建设电梯安全应急服务平台，启动国家危险化学品应急救援基地建设。科学组织、全力保障，有力应对近40年来最大汛情，最大限度减轻地质灾害危害，避险转移群众20余万人，全市无人因洪涝、地质灾害死亡。每月开展城市安全风险分析预警，实施“9+3”安全生产专项整治，安全生产事故和死亡人数分别下降2.9%、14.4%。持续推进网络理政。优化提升网络理政“11637”架构体系，启动建设城市智慧治理中心，加强政府网站建设，市政府网站获评全国省会城市第一。完成市级政务云平台搭建，641个应用系统迁移上云。“天府市民云”APP上线运行。699项政务服务事项可在网上办理，91%的审批服务事项实现“最多跑一次”。建成全市统一综合行政执法智慧服务平台。网络理政平台受理群众来电来信318万件，诉求解决满意率88.8%，被评为全国“互联网+政务”领域十佳优秀案例。网络理政已成为访民意、知民情、解民忧的最短路径！

（四）优化基本公共服务。强化设施配套。实施高品质公共服务设施攻坚计划，扎实推进中心城区公共服务设施“三年攻坚”行动，深入落实“818”专项规划，开工建设902个项目、建成350个项目，累计投资104亿元。深入推进“厕所革命”，新改建公厕700座。开展老旧小区加装电梯试点。新建环卫工人作息房166个。攻坚克难、稳妥推进，群众反映强烈、拖了8年多的3508厂自主搬迁重大民生项目取得明显进展。扎实推进就业社保工作。抓好重点群体就业创业，动态消除“零就业”家庭，新增城镇就业27.6万人，城镇登记失业率保持在4%以内。深入推进居家和社区养老服务改革试点，新建社区养老院61家、城乡社区日间照料中心207个。关心关爱残疾人，提供服务749万项次。配租配售保障房2.3万套。城乡居民基本养老保险参保率保持在90%以上，基本医疗保险参保率达98%。城乡低保补助水平分别增长19.8%、27%。退役军人安置、管理和保障工作扎实有力。大力发展教育事业。全面推进国家级教育改革示范试点。新投用幼儿园98所、中小学55所，分别新增学位3万个、8.2万个，公办和公益性幼儿园学位覆盖率达到87.3%。全面消除超大班额，37.8万名流动务工人员随迁子女在蓉接受义务教育。特殊教育、社区教育、职业技术教育水平不断提升。大力支持在蓉高校“双一流”建设。切实提升公共文化服务。公共图书馆、文化馆、乡镇（街道）和村（社区）综合性文化服务中心全部免费开放。开展文化惠民活动7万余场。加快建设健康城市。新增三级甲等综合医院5所。实现医联体基层全覆盖，459个医疗机构互认检验结果。61种治疗重特大疾病的药品纳入医保支付。2万余名重度失能人员享受长期照护保险。广泛开展全民健身活动，全市人均期望寿命达80.54岁，被评为2018健康中国年度标志城市。加快构建公共法律服务体系。实现四级公共法律服务实体平台全覆盖，建立“成都法网”网络平台，为群众提供法律援助12.3万人次。全面完成十大民生实事。连续10年位居“中国最具幸福感城市”榜首！

（五）扎实推进生态环境保护。持续打好大气、水、土壤污染防治“三大战役”，切实抓好“三治一增”，实施突出环境问题整治“百日攻坚”专项行动。扎实开展环保督察及“回头看”反馈问题整改。有序推进第二次全国污染源普查工作。深入实施“治霾十条”和大气污染防治“650”工程。累计完成大蒸吨燃煤锅炉清洁能源改造911台，餐饮门店清洁能源改造入选联合国教科文组织推广案例，新增充换电站112座、充电桩4205个，中心城区新增公交全部使用新能源车，全市清洁能源占比达58.8%。强化科技治霾和各类扬尘治理，在全国率先实施非道路移动机械备案登记和标志管理，PM10、PM2.5、O3平均浓度分别下降8%、8.9%、2.3%，空气质量优良天数增加16天、重污染天数减少16天。深入实施“治水十条”和水污染防治“626”工程。全面落实河湖长制，加快实施锦江、沱江、金马河流域水生态治理，基本消除城市建成区黑臭水体，106个地表水断面中I至III类水质占比提高7.2个百分点，国控锦江黄龙溪出境断面水质从V类提升到IV类。深入实施“治土十条”和土壤污染防治“620”工程。开展土壤环境质量调查，加快建设45个固体废物污染防治项目。农药、化肥使用量分别减少2%、1.5%。深入开展全域增绿。加快大熊猫国家公园建设和大地景观塑造，龙泉山城市森林公园“增绿增景”6.5万亩，全市森林覆盖率达39.5%。开展城区绿化春季、秋季战役，新建公园、小游园和微绿地188个，新增绿地670万平方米。国家低碳试点城市建设取得成效。大力推进低碳能源、产业、消费、交通、城建和碳市场体系建设，发布国内首个城市低碳发展蓝皮书，承办第二届国际城市可持续发展高层论坛。长安静脉产业园成功创建国家资源循环利用基地。万元地区生产总值能耗降低5%，荣获“全球绿色低碳领域先锋城市蓝天奖”。这一年，通过全市上下共同努力，实现空气质量优良天数251天，达到新标准实施以来最好水平，锦江水生态治理获习近平总书记充分肯定！

（六）不断深化双向开放合作。深度融入“一带一路”建设和长江经济带发展，积极推进“四向拓展、全域开放”，加快打造国际门户枢纽。不断拓展大通道。新开通国际定期直飞客货运航线12条，国际（地区）航线达114条。加快建设天府国际机场，累计完成投资204亿元。双流国际机场旅客吞吐量达5295万人次，其中国际（地区）旅客599.8万人次，位居全球最繁忙机场26位。新开通“蓉欧+”东盟铁海联运班列和铁路班列，国际班列通达24个海外城市，蓉欧班列全年开行1591列、开行量继续保持全国第一。加快搭建大平台。天府新区成都片区保税物流中心（B型）、高新西园综保区获批建设，中国—欧洲中心正式运营，中国（四川）—东盟自贸合作中心成立。自贸区新增注册企业4.8万户，新增注册资本7008.5亿元。全球跨境电商服务资源中心、国际商事纠纷调解中心正式运营。国别合作园区加快建设，累计签约引进项目124个。落户世界500强企业达285家。奥地利驻成都总领事馆正式开馆，驻蓉领事机构达17个。“海外成都”工作站达20个。加快发展大经贸。积极参加首届中国国际进口博览会。获批深化服务贸易创新发展试点和市场采购贸易方式试点。新备案境外投资企业和机构118家。组织赴70多个国家开展投促活动。开展投资促进“百日擂台赛”，全年签约引进重大项目420个，实际到位内资5650亿元，利用外资实际到位120亿美元。实现外贸进出口总额4983.2亿元、增长26.4%。不断深化大交流。成功承办第二届中国—中东欧国家艺术合作论坛、第四届阿拉伯艺术节，成功举办2018欧洲文化季、国际友城青年音乐周等国际交流活动，国际友城和友好合作关系城市达93个。设立“一带一路”留学生政府奖学金。获批实施部分国家外国人144小时过境免签政策。深入推进区域合作。认真落实“一干多支、五区协同”发展战略，签署成德眉资同城化合作协议，推动环成都经济圈7市一体化发展，与20个市州分别达成全面合作协议。开展“成都企业市州行”，签署投资协议183个、总投资932.1亿元。加快成渝城市群一体化发展，与广西开展全方位合作，加强与京津冀、粤港澳大湾区、长三角等地区交流合作。成都的朋友圈儿越来越大，朋友越来越多！

（七）大力彰显天府文化魅力。高水平建设世界文创名城。编制文创聚集区规划，新增市级文创产业园区31个，文创产业增加值超过1000亿元。高标准建设世界赛事名城。规划天府奥体城，开工建设凤凰山体育中心、皇冠湖体育中心，成功举办ATP250成都网球公开赛、都市自行车世界锦标赛等国际体育赛事21个。高标准筹备第十八届世界警察和消防员运动会，成功申办第三十一届世界大学生夏季运动会。高品质建设国际音乐之都。城市音乐厅、露天音乐公园、新声剧场建成试运行。新培育引进音乐企业103家，举办各类音乐演出1600余场，支持和规范街头艺术表演，音乐产业产值增长21.7%。加强历史文化遗产保护。全面推进文物保护基础工作，完成东华门、体育中心遗址考古发掘，都江堰水利工程列入世界灌溉工程遗产名录，新增历史保护建筑63处。拓展文化对外交流。成都大学中国—东盟艺术学院正式招生。摩洛哥拉巴特中国文化中心揭牌运营。成功承办第十三届孔子学院大会、举办世界文化名城论坛天府论坛等文化交流活动30余场。天府文化正加快走向世界，成为彰显中国气派、巴蜀魅力、成都精神的独特标识！

（八）深入实施乡村振兴战略。扎实推进“十大重点工程”“五项重点改革”，编制完成乡村振兴战略规划、空间发展规划和乡村振兴示范走廊规划，提升建设服务全川“七大共享平台”，策划推出重大项目586个、完成投资614.6亿元。切实巩固脱贫攻坚成果。持续深化“回头看”“回头帮”，建档立卡贫困户人均可支配收入增长15%。加大对口支援甘孜、阿坝深度贫困县精准脱贫工作力度，实施援助项目495个，投入援助资金4.9亿元。支持九寨沟灾后恢复重建。持续深化农业农村改革。获批“探索建立新型职业农民制度”等3项全国农村改革试点，全面完成农村集体资产清产核资，持证农业职业经理人达1.2万人，土地适度规模经营率达65.3%。强化农村要素保障。“农贷通”平台放款58.3亿元。启动建设国家成都农业科技中心。打造简阳、邛崃国家级返乡创业示范园区。大力发展农村电商，农产品网上交易额达87.8亿元、增长46%。加快建设美丽乡村。建立特色镇规划建设标准体系，启动57个特色镇培育创建。规划建设天府农耕文明博物馆。新改建农村公路218公里，启动农村户厕改造9万余户。全面加强农业面源污染治理。保护修复川西林盘100个。蒲江县明月村获评“2018十大中国最美乡村”，郫都区战旗村获评“中国美丽休闲乡村”。全省乡村振兴现场会在蓉召开，成都为乡村振兴四川篇章交出了优异答卷！

（九）全面提升城市功能品质。优化城市空间布局。深入实施“东进、南拓、西控、北改、中优”，全面落实“五中心一枢纽”建设6个专项行动计划。加快城市总规修编，高水平规划南北、东西城市轴线，制定龙泉山东侧沱江发展轴总体规划初步方案。加快建设李家岩水库等重大工程。加快建设美丽宜居公园城市。编制美丽宜居公园城市规划及建设导则，成立全国首个公园城市研究院，启动鹿溪智谷、交子公园、天府锦城三大示范工程。熊猫绿道建成开放，锦江绿道、锦城绿道加快建设，建成天府绿道2607公里。加速推进“三铁融合”。成蒲铁路、川藏铁路（朝阳湖至雅安段）建成投用，成兰、成贵铁路加快推进。成德、成绵、成灌、成彭动车加密开行，成都西站建成投用。地铁1号线三期、3号线二三期、有轨电车蓉2号线开通运行，城市轨道交通累计运营里程240公里，地铁在建里程283公里。加快编制TOD战略规划，启动13个示范项目。完善市域交通体系。成资渝、成宜高速开工建设，天府国际机场高速加快推进。成资、成龙简、金简仁等东部新城骨干道路，龙泉山城市森林公园旅游环线、天府大道北延线等开工建设。打通东泰路等“断头路”80条。慢行交通体系持续优化。全国首条封闭式旅游自行车高速路在邛崃建成投用。中心城区新开公交线路59条、夜间巴士线路14条。强化城市精细化管理。制定城市精细化管理标准，开展共享单车集中规范整治。完成1918条背街小巷提升整治和109条特色街区打造，开展“最美街道”“最差街道”评选。生活垃圾分类覆盖率达35.2%。改造棚户区1.2万户、老旧院落327个。强力推进“两拆一增”，拆除违法建（构）筑物136万平方米，拆除围墙31万米，增加透绿开敞空间480万平方米，城市空间更敞亮，绿色与我们更亲近！

（十）持续全面推进改革创新。以“五项制度改革”为重点，推动高质量发展。持续推进供给侧结构性改革。扎实开展“三去一降一补”，认真落实国家减税降费系列政策和电、天然气优惠政策。取消“五路一桥”收费，减少市民费用12亿元。持续深化“放管服”改革。深入开展减证便民专项行动，行政机关、村（社区）证明事项分别减少至12项、15项。开展工程建设项目审批制度改革试点，全流程审批总用时压缩至120个工作日内。公共资源交易平台实现一体化运行和全流程电子化交易。初步完成“11+2”中心城区管理体制改革。深入推进商事制度改革。全面推开“证照分离”改革，实现涉企证照“四十三证合一”，推广全程电子化网上登记。实施企业名称网上自主申报，企业开办时间压缩至5个工作日内。加快推进国资国企改革。积极推进以效率为导向的国资经营评价制度改革，突出“两降两提”要求，关停“僵尸企业”和低质低效企业110户，市属国企资产总额增长18.1%，证券化率达48.8%。深入推进财税体制改革。积极推进以绩效为导向的财政预算制度改革，中心城区新财政体制平稳运行，地方政府债务风险持续降低。完成国税地税征管体制改革阶段性任务，社保费和非税收入征管职责划转有效推进。基本完成市属企事业单位公务用车改革。深入推进全面创新改革。国务院先后在全国复制推广的36项全面创新改革经验中，9项由成都探索形成。完成近400项职务科技成果分割确权，建成国家技术转移西南中心，获批国家首家新药专项成果转化示范基地。中科院成都科学研究中心、西南地质科技创新中心等项目开工建设，建成各类产学研创新平台1200个。获批成德绵国家科技成果转移转化示范区。成都知识产权交易中心正式运营。全社会研发投入增长12%，每万人有效发明专利拥有量22件，获年度国家科学技术奖29项。高新技术企业超过3000家，高新技术产业总产值突破1万亿元。深入推进军民融合发展，获批国家首批知识产权军民融合试点、省低空空域改革试点，军民融合产业主营业务收入增长14%。扎实推动大众创业、万众创新。成功举办第四届全国“双创”活动周主会场活动、成都全球创新创业交易会。新建创新创业载体36家、51万平方米，开展菁蓉汇品牌系列活动168场。新增市场主体54万户，近三年年均增长28%。深入落实“人才新政12条”，新引进国家“千人计划”专家34人，累计吸引超过25万名本科及以上学历毕业生落户。成都已成为全国创新创业活跃、年轻人向往的热土！

（十一）切实加强政府自身建设。落实全面从严治党主体责任。严明政治纪律和政治规矩，牢固树立“四个意识”，切实增强“四个自信”，坚决做到“两个维护”，扎实推动中央、省委巡视反馈问题整改落实。推进“两学一做”学习教育常态化制度化，深入开展“大学习、大讨论、大调研”。认真贯彻党风廉政建设各项规定，大力整治群众身边的不正之风和“微腐败”。深入推进依法行政。严格执行市人大及其常委会决议决定，主动接受法律监督和工作监督，自觉接受市政协民主监督，办理人大代表建议573件、政协提案667件，办复率100%、满意率达99.8%。健全权力清单和责任清单动态调整机制，向社会公开5925项市级部门行政职权事项。严格落实重大行政决策程序和“三重一大”事项决策机制，制度性开展市政府常务会会前学法。加强政务诚信建设。建立健全政务诚信管理运行、监督考核机制，强化招商引资、企业服务等重点领域政务诚信建设。建立全市统一政务数据共享交换平台和市公共数据开放平台，完成基层政务公开标准化规范化建设，政务公开全面深入开展。

国家安全、国防动员、防灾减灾、应急管理、消防、人防、双拥工作取得新进展，审计、统计、档案、保密、气象、邮政、参事、地方志、哲学社会科学等工作取得新成绩，民族宗教、港澳台、妇女儿童、青少年、老龄、慈善等工作取得新成效。

各位代表，过去一年，在国际形势复杂多变和经济发展面临多重困难的情况下，全市经济社会保持稳中有进、稳中向好态势，成绩来之不易！这是坚持以习近平新时代中国特色社会主义思想为指导，认真贯彻落实党中央国务院、省委省政府和市委决策部署的结果，是全市人民齐心协力、奋力拼搏的结果。在此，我代表市人民政府，向全市人民，向人大代表、政协委员、各民主党派、工商联、无党派人士、各人民团体，向中央和省驻蓉单位，向所有关心支持成都建设发展的同志们及各界人士，表示衷心的感谢！向人民解放军指战员、武警官兵、政法干警和民兵预备役人员，表示崇高的敬意！向港澳台同胞、广大侨胞和海内外朋友们，表示诚挚的谢意！

我们还清醒认识到，对表新发展理念，当前工作仍然存在不少问题、面临诸多挑战：自主创新能力不强，激励企业加大研发投入的政策还不多，企业研发投入偏低，军工和科技资源优势发挥不够。城乡发展、区域发展协调性还需进一步提升，各区域优化功能的力度还不够大，乡村全面振兴还需持续用力，“主干”引领辐射带动作用还需加强。经济社会绿色低碳转型还需加速，生态环境保护任务依然繁重，大气、水、土壤污染治理难度加大，绿色低碳生产生活方式尚需加强引导。双向开放水平还需提高，吸纳聚集全球优质资源要素的能力还不强，国际化营商环境还不优。发展成果共享的力度还需加大，幼儿园、中小学学位还显不足，医疗资源分布还不均衡，养老服务设施相对滞后，推动共建共治共享美好家园的措施还不够。针对这些问题，我们将以更大的决心、更有力的举措，切实加以解决。

二、2019年工作安排

2019年是新中国成立70周年，是全面建成小康社会关键之年。今年工作的总体思路是：坚持以习近平新时代中国特色社会主义思想为指导，深入学习贯彻习近平总书记对四川及成都工作系列重要指示精神和中央经济工作会议精神，全面贯彻党的十九大和十九届二中、三中全会精神，省委十一届三次、四次全会和省委经济工作会议，市委十三届三次、四次全会暨市委经济工作会议决策部署，坚持稳中求进工作总基调，坚持新发展理念，坚持以供给侧结构性改革为主线，坚持深化改革开放，推动形成“一干多支、五区协同”新格局、“四向拓展、全域开放”新态势，统筹推进稳增长、促改革、调结构、惠民生、防风险工作，进一步稳就业、稳金融、稳外贸、稳外资、稳投资、稳预期，全面贯彻“巩固、增强、提升、畅通”八字方针，切实履行政府职能，扎实开展国际化营商环境建设年工作，全面推动高质量发展、实施高效能治理、创造高品质生活，加快建设全面体现新发展理念的国家中心城市。

今年发展的主要预期目标是：地区生产总值增长7.5%左右，全社会固定资产投资增长10%，社会消费品零售总额增长9%，一般公共预算收入与经济保持同步增长，城乡居民人均可支配收入分别增长7.5%、8%，全社会研发投入增长12%，万元地区生产总值能耗、主要污染物排放进一步降低，城镇登记失业率控制在4%以内。重点抓好七个方面工作：

（一）聚力推动经济高质量发展。坚持走产业结构优、科技含量高、资源消耗少、环境影响小、质量效益好、发展可持续之路，加快实现高质量发展。

做强做优先进制造业。深入推动66个产业功能区建设，提升产业承载和吸引能力。高标准建设标准厂房800万平方米、园区配套住房400万平方米。做强现代产业体系，加快打造全国电子信息、高端装备制造基地和软件名城，力争电子信息产业规模超过8000亿元、装备制造产业规模超过7000亿元。推动医药健康产业持续快速发展，力争产业规模超过4000亿元。规模以上工业增加值增长7.8%。强化项目支撑，围绕做大做强产业集群，大力引进一批重大工业项目。推动海思科生命科技产业基地、高通量宽带卫星产业基地等120个项目开工建设。促进紫光成都存储器基地、京东方第6代低温多晶硅生产线等项目加快建设。推动吉利BMA乘用车平台、威高医疗装备产业园等130个项目竣工投产。强化要素保障，帮助重点企业稳产达产。促进大中小企业融通发展。推动先进制造业和现代服务业深度融合。加快发展电子商务、工业设计、技术咨询、检验检测认证等生产性服务业，推进工业互联网应用和智能制造，促进工业绿色低碳发展。

培育壮大消费市场。努力建设国际消费中心城市，加快口岸进境免税店、免税购物中心、区域性进口商品展示交易中心建设，支持蜀锦、蜀绣、漆器等成都特色工艺与国内外知名品牌合作，加大老字号消费品牌国际推广力度。深化“品质成都”建设，加快五大新兴服务业提挡升级，大力提升“成都制造”“成都创造”“成都服务”品质。改善消费环境，提升打造春熙路、锦江夜游等消费商圈，提升建设太古里、宽窄巷子等国家级步行商业街示范街区。创新消费模式，完善定制消费、医美消费等新型消费服务功能，支持新零售创新发展，努力满足多元化消费需求。全面落实个人所得税专项附加扣除政策，不断激发居民消费潜力。

加快发展新经济培育新动能。促进数字经济与实体经济深度融合，大力推动5G、8K、人工智能、物联网、网络安全、氢能等全产业链发展。深化新经济企业梯度培育计划和“双百工程”，发布“城市机会清单”，推进成都造“首台套”示范应用。完善新经济天使投资基金、知识产权运营基金管理，推动优秀科技企业在科创板上市。力争新增独角兽企业2家以上，新经济产值增长18%。

支持民营经济持续健康发展。大力推动“民营经济25条”落地落实，深入推进以创新和成长为导向的企业扶持激励制度改革。支持本土企业做大做强，促进中小企业“专精特新”发展，培育更多细分行业的“单打冠军”。强化金融服务实体经济能力，建立“5+2”全生命周期的投融资服务体系，吸引更多风投、创投机构聚集。深入实施“交子之星”上市倍增行动、债券优先发展计划，力争新增上市公司8家。依法保护企业家财产权、创新权益和自主经营权，持续开展解决民营企业历史遗留问题专项行动。大力弘扬“蓉城企业家”“蓉城工匠”精神。增强服务企业就是服务发展的意识，真心真情服务企业，构建亲清新型政商关系！

（二）聚力全面深化改革创新。坚持向改革要红利、向创新要活力，将改革创新作为推动发展的不竭动力。

深入推进创新驱动发展。加快建设国家自主创新示范区，争取一批国家级创新基地和项目落户，加快建设成都超级计算中心。加快成德绵国家科技成果转移转化示范区建设，深入实施蓉城科技聚变计划，扩大职务科技成果权属混合所有制改革应用范围及成效，深化环高校知识经济圈建设，力争国家高新技术企业达3300家，每万人有效发明专利拥有量23件。规划建设军民融合成果转化交易中心，积极参与创建国家军民融合创新示范区，深化知识产权军民融合试点建设。高质量推进人力资源提升行动，深入实施“蓉漂计划”、本土人才培育计划，大力培育产业工人队伍。深入开展菁蓉汇、“蓉”归故里系列活动，加快国家双创示范基地建设，高水平举办第五届成都全球创新创业交易会。

扎实开展重点领域改革。深入推进“五项制度改革”，提高资源配置效率、提升公共服务质量。加快转变财政投入方式，全面实施预算绩效管理。深入推进国资国企改革，持续深化“三项制度”和专业化公司改革，加快完善市场化经营机制和以管资本为主的监管体系，新增国企上市或挂牌企业4户以上。深入推进土地资源配置制度改革，形成分区域、分性质、分行业的投资门槛标准。开展基本公共服务标准化试点，加快完善政府购买公共服务机制，优化公共服务供给结构。持续推进税收征管体制改革。健全举债融资机制，防范化解政府隐性债务风险。稳妥推进住房公积金制度改革。构建地方金融“监管沙箱”，切实提升地方金融风险防控水平，加快建设西部金融中心。

着力优化营商环境。对标世界银行营商环境评估标准和国家营商环境评价体系，全面提升市场投资、政务服务、科研创新、人才要素、法治保障等环境。加快落实国家减税降费政策，切实降低市场主体营商成本。深入推进“放管服”改革，深化“最多跑一次”改革。继续推行企业投资项目承诺制，全面深化工程建设项目审批制度改革，力争将全流程审批用时压缩到90个工作日内。继续推进公共服务类行政事业单位错时延时服务。深化商事制度改革，扩大全程电子化登记的业务类型。加快社会信用体系建设，健全“红黑名单”制度。加快建设“一带一路”国际仲裁中心、国际商事纠纷调解中心、外国法查明中心，推行外商投资企业商务备案与工商登记“单一窗口、单一表格”办理，提升外商投资便利度。

（三）聚力建设美丽宜居公园城市。突出公园城市特点，把生态价值考虑进去，加快塑造人城境业和谐统一的大美公园城市形态。

优化城市空间布局。深入推进“东进、南拓、西控、北改、中优”，积极构建“一心两翼三轴多中心”城市空间结构。加大“东进”力度，坚持精筑城、广聚人、强功能、兴产业，突出重点、循序渐进、稳步推进，打造龙泉山东侧沱江发展轴，加快空港新城、天府奥体城、国际高等教育园区建设，推进简州新城、淮州新城、龙泉山城市森林公园建设，加快建设地铁18号线和成资、成龙简快速路等基础设施，强力推进大运村、东部新城市民中心、金堂通用航空机场等重大支撑性项目建设。“南拓”区域加快建设天府国际会议中心、天府中心、鹿溪智谷、独角兽岛。“西控”区域加快大熊猫国家公园、李家岩水库建设，推进都江堰精华灌区修复工程和川西林盘整治提升。“北改”区域强化国际铁路港门户枢纽和公铁联运国际物流枢纽功能。“中优”区域加快天府锦城、交子公园等重大工程建设。

加快生态环境建设。打好污染防治攻坚战，持续实施“治霾十条”和大气污染防治“650”工程，强化科技治霾成果推广应用，持续开展夏季臭氧防控专项行动、秋冬季大气污染防治攻坚行动，努力提升空气质量。持续实施“治水十条”和水污染防治“626”工程，继续推进锦江、沱江、金马河流域水生态治理，完成城市建成区外123段黑臭水体治理，新建污水管网460公里，生活污水集中处理率达到91%。持续实施“治土十条”和土壤污染防治“620”工程，深入推进固体废物污染防治三年攻坚，持续减少农药、化肥使用量。扎实开展噪声、光污染等综合整治。推进全域增绿，加快建设天府绿道、天府植物园和“百个公园”示范工程，新建成绿道800公里，森林覆盖率达到39.9%。大力推广立体绿化，加快城市森林花园建筑和花园式特色街区建设。持续提升清洁能源使用率。继续抓好国家低碳试点市建设，积极创建国家生态文明建设示范市，推动形成绿色低碳的生产生活方式，让天府成都青山常在、绿水长流、空气常新！

加快完善城市配套。开通试运营地铁5号线一二期、10号线二期，加快6号线、8号线、9号线、17号线等地铁项目建设。科学布局公交线网。加快推进南北、东西城市轴线建设，完善中心城区“1环11射”快速路。加快完善行人、非机动车和公交车优先的路权体系，不断优化慢行交通系统。实施科学治堵，构建智慧交通体系，力争全市交通拥堵指数不上升。大力实施中心城区公共服务设施“三年攻坚”行动，新开工项目800个以上、建成项目350个。开工建设13个TOD综合开发示范项目。

加快提升城市品质。编制完成社区发展规划，推动“智慧养老”“智慧医疗”“智慧物业”等服务进社区，打造高品质和谐宜居生活社区。大力推进智慧城市建设，建成并运行城市智慧治理中心，构建智能城市指挥运行体系，把握5G试点和AI应用机遇，打造全天候能在线监测、能分析预测、能应急指挥的“城市大脑”。深入推进城市精细化管理，全域推进“两拆一增”，加快背街小巷环境提升，加强城郊结合部环境综合整治。继续打通“断头路”。深入实施“厕所革命”，新改建公厕608座。优化城市景观照明。制定城市道路环卫作业及维护施工安全保障办法、环卫工人安全健康关爱办法，持续新建环卫工人作息房。推动出台生活垃圾管理条例，积极推进垃圾分类。

（四）聚力打造国际门户枢纽。深度融入“一带一路”建设和长江经济带发展，坚持“四向拓展、全域开放”，加快打造国际门户枢纽和内陆开放高地。

加快建设大通道。加快天府国际机场建设，提升双流国际机场枢纽功能，加快布局“48+14+30”的国际航空客货运战略大通道，新增国际定期直飞航线5条以上。加密国际铁路通道网络，加快班列海外枢纽节点建设，积极参与国际陆海贸易新通道建设。推动成都至自贡高铁开工建设、沿江高铁成达段启动前期工作，全力服务川藏铁路规划建设。加快天府国际机场高速、成资渝高速、成都经济区环线高速公路等项目建设。大力推进天新邛、成彭等快速路建设。加快成都国际铁路港建设，深化多式联运“一单制”等改革，提升集散分拨能力和通关服务水平，做强国际多式联运体系。

健全完善大平台。加快自贸试验区建设。推动双流国际机场空运进口肉类指定口岸、铁路进境粮食指定口岸验收投运，高新西园综保区、天府新区保税物流中心（B型）封关运行。推动货物和服务贸易协调发展，深入推进市场采购贸易方式试点，大力推广应用国际贸易“单一窗口”。加快“一带一路”临港产业合作园规划建设。积极争创内陆自由贸易港。加快已有国别合作园区建设，力争引进项目160个，推动中日产业合作园落地。加快中国（四川）—东盟自由贸易合作中心、“一带一路”大厦建设。推动设立成都国际铁路港国家级经济技术开发区和综合保税区，规划建设“金青新大港区”。

加快推动大经贸。加快引进跨国公司区域总部、国际中小企业，力争利用外资实际到位130亿美元以上。持续开展投资促进“百日擂台赛”，力争全年新签约引进重大项目440个，到位内资6300亿元以上。支持优势产业、优秀企业、优质产品“走出去”，制定“一带一路”重点国家《国别投资指南》，鼓励企业建设境外产业合作园区。进出口总额增长5%。

持续深化大交流。吸引更多国家在蓉设立领事机构，推动与更多城市建立国际友城或友好合作关系，吸引更多国际机构、经贸和旅游代表处等落户。深入实施外籍人士“家在成都”工程，积极推动“侨之家”建设，加快建设国际化社区。打造“留学成都”品牌，提高成都大学中国—东盟艺术学院影响力，全面实施部分国家外国人144小时过境免签政策。深化低碳领域国际合作，推进中瑞低碳城市二期合作项目，加强与C40等国际组织交流。积极推动与京津冀、粤港澳大湾区、长三角、泛珠三角等地区合作。

发挥主干引领辐射带动作用。深入落实与省内市州全面合作协议、部门及企业系列合作专项协议，构建“总部研发、生产配套”新型合作关系，持续开展“成都企业市州行”。深入推进成德眉资发展规划、基础设施等同城化。有序推动成都平原经济区一体化发展，加快推动铁路公交化运营，打造“1小时”城际综合交通运输网络。加快推进五区协同发展，加强与泸州港、宜宾港互动合作，深化自贸试验区三大片区联动。加快推进省内市州合作园区建设。

（五）聚力实施乡村振兴战略。统筹推进“五个振兴”，抓好重点改革，实施好重点工程，努力在乡村振兴中走在前列、起好示范。

加快培育乡村产业新优势。大力发展都市现代农业，加快建设“6+7”现代农业产业功能区（园区），新建高标准农田30万亩，牢牢守住粮食安全底线。加快建设国家现代农业产业科技创新中心、国家成都农业科技中心。加快推动乡村产业跨界融合，启动“农村集体经济+农商文旅体融合发展”试点项目30个。大力发展农产品精深加工，完成“三品一标”认证15个以上，提升“天府源”品牌价值。完善“七大共享平台”，全面深化农业区域合作。力争农业增加值增长3%。

充分激发农村改革新动能。深化农村集体资产股份化改革，健全农村产权交易服务机制，规范开展农村集体经济组织登记赋码。鼓励引导各类人才返乡创业，引进培育一批新型职业农民和乡村急需紧缺高端专业人才。力争农业适度规模经营率达到70%。提升建设“农贷通”、益农信息社平台，启动中药材保险试点。深化财政支农资金形成资产股权量化改革。

加快打造幸福美丽新乡村。建设300个川西林盘，打造20个特色镇（街区）。加快建设“四好农村路”，新改建农村公路200公里以上。完成1.2万户农村土坯房改造。广泛开展文明村镇创建和乡村文脉保护传承行动。强化现代信息与技术服务。实施农村人居环境整治提升“百镇千村”攻坚行动，完成农村户厕改造提升24.8万户，培育“美丽四川·宜居乡村”达标村1500个。

（六）聚力建设世界文化名城。坚定文化自信，大力传承巴蜀文明、发展天府文化，凝炼城市时代精神，不断提升天府文化影响力。

强化文化遗产保护利用。全面加强历史遗址、历史街区、历史建筑和名人故里、工农业遗产等科学保护利用。加快宝墩国家考古遗址公园等建设。系统梳理古蜀、三国、水利、诗歌等历史文化资源，全力推进李冰、诸葛亮、杜甫等历史名人“六个一批”建设。实施天府手艺振兴行动，把民间习俗、民间手艺等融入日常生活，实现活态传承。办好第七届中国成都国际非物质文化遗产节。

加快塑造“三城三都”城市品牌。贯彻落实加快建设世界文化名城的决定。力争服务业增加值增长8.6%。加快建设文创名城，推动熊猫、川剧、川菜等文化的衍生品创新，打造更多“成都礼物”精品，力争文创产业增加值增长15%。加快建设旅游名城，充分挖掘古蜀、熊猫、休闲、绿道和美食文化潜力，丰富夜游经济，塑造核心旅游品牌，推出更多精品旅游线路，推进国家全域旅游示范区和天府旅游名县建设，开展“南方丝路·熊猫之都”主题营销，力争入境游客380万人次，旅游总人数2.6亿人次、总收入4650亿元。加快建设赛事名城，高水平建设皇冠湖体育中心、凤凰山体育中心，高质量办好第十八届世界警察和消防员运动会、2019年乒乓球世界杯赛等22项国际体育赛事，高标准筹备第三十一届世界大学生夏季运动会，积极申办2022年世界乒乓球锦标赛、2025年世界运动会，以筹办重大体育赛事为抓手，推动各类配套设施加快建设，提升市民生活品质和城市形象。加快建设美食之都，引进一批国际知名餐饮品牌企业，继续推行餐饮标准化管理、精细化标示，举办海外美食行、亚洲美食节等活动。加快建设音乐之都，全面提升城市音乐厅、露天音乐公园建设运营水平，推动东郊记忆音乐公园、成都音乐坊等提挡升级，支持特色音乐小镇差异化发展，高水平办好中国音乐金钟奖等国内外知名音乐赛事和活动，力争音乐产业保持快速增长。加快建设会展之都，持续引进国际品牌展会，办好第六十届泛美开发银行理事会年会、公园城市全球论坛等重大会展活动850个，实现会展业总收入1300亿元。

推动天府文化走向世界。加快成都自然博物馆、成都金融城文化中心等重大文化地标建设。建立天府成都国际传播研究中心和城市宣传海外基地。梳理世界名人在蓉足迹，讲好世界名人的“成都故事”。高水平举办国际诗歌周、国际童声合唱音乐周等国际文化交流活动。

（七）聚力提升市民获得感幸福感安全感。努力满足人民群众美好生活需要，解决市民“急难愁盼”，全力促进“有感发展”。

巩固提升脱贫攻坚成效。细化落实“五大提升行动”，进一步提升脱贫攻坚质效。改善经济薄弱村公路通行条件和医疗卫生保障水平。强化对巩固提升重点户、产业帮扶户、经济薄弱村的产业帮扶，发展壮大集体经济，提升脱贫造血功能。持续提高贫困人口多元就业能力，强化城市相对贫困人口帮扶。加大经济薄弱村新型职业农民培育力度，引导“新村民”和各类专业人才集聚。继续做好援藏帮彝、定点扶贫、九寨沟灾后恢复重建帮扶工作。

大力发展民生社会事业。继续抓好十大民生实事。高质量发展教育事业。新建、改扩建幼儿园60所、中小学40所，分别新增学位2万个、5万个。全面落实教育助学政策，做好流动务工人员随迁子女入学工作。提升社区教育服务品质，大力发展职业技术教育。支持和规范社会力量兴办教育。加大与在蓉高校“双一流”共建力度，高水平办好成都大学等市属高校。引进更多国际优质教育资源。丰富公共文化服务供给。加快推进公共文化服务标准化、均等化，深入实施文化惠民工程，提高基层公共文化设施综合使用效率。提升就业社保服务水平。加强高校毕业生、退役军人、去产能职工等就业服务。深入实施全民技能提升计划，健全终身职业技能培训制度，深化社会援助和托底帮扶，实现城镇新增就业23万人。完善城乡社会保障制度体系，深入实施全民参保计划，落实降低社保费率政策。提高城乡低保和特困人员救助供养标准。重点推进居家养老、社区嵌入式养老，加快推进老旧社区加装电梯，设置老年人助餐服务点200个以上。加快发展卫生健康事业。积极创建国家健康城市示范市，争取国家“优质医疗资源扩容下延工程”试点。大力发展中医药事业。深入推进全民健身，创建全民运动健身模范市。强化留守儿童、空巢老人关爱保护，持续加强城市一线管理服务人员关心关爱。坚持“房子是用来住的、不是用来炒的”定位，构建房地产市场健康发展长效机制，完善住房保障体系，按照产城相融、职住平衡理念，积极解决好住房“刚需”“刚改”问题，努力满足市民多元化住房需求。

深入推进市场监管。深化市场监管体制改革，加快构建现代化市场监管体系。深化“双随机、一公开”监管，推进线上线下监管一体化。加强专利权、商标权、版权等要素市场和产权市场法治建设。深入推进食品安全示范城市创建，加强食品药品智慧监管，打击网络非法经营等违法犯罪行为。加强重点工业产品和日用消费品监督抽查。加强特种设备安全监管和隐患排查治理，完善电梯应急服务平台。加强价格监管和调控，居民消费价格上涨控制在3%以内。

不断提升社会治理水平。拓展优化网络理政“11637”体系功能，深化智慧治理。深化“一核三治，共建共享”新型社区治理机制，深化街道（乡镇）、社区（村）减负赋能增效。深入推进“法律七进”，完善公共法律服务体系。做好信访工作，强化“群众工作之家”建设。加强国防动员工作和后备力量建设，深入开展双拥共建活动。大力推进平安成都建设，推动扫黑除恶专项斗争向纵深发展，系统打击、综合治理突出违法犯罪。完善风险防控机制，积极防范化解重大风险。全面推进国家安全发展示范城市创建，强化安全风险管控和隐患排查治理，健全应急管理体制机制，提升抵御自然灾害和防灾减灾救灾能力，确保城市安全、社会安定、市民安宁！

三、全面加强政府自身建设

牢固树立“四个意识”，切实增强“四个自信”，坚决做到“两个维护”，坚持以人民为中心的发展思想，加快建设新时代人民满意政府。

（一）加强政府系统党的政治建设。高举习近平新时代中国特色社会主义思想伟大旗帜，学习贯彻习近平总书记重要讲话精神和党中央国务院、省委省政府、市委重大决策部署，常态化制度化开展“两学一做”学习教育。认真落实全面从严治党主体责任，严明政治纪律和政治规矩，推动巡视反馈问题整改落实。持续抓好党风廉政建设，认真落实“一岗双责”，严格落实中央八项规定及实施细则精神和省市实施细则要求，加强廉政风险防控，系统治理基层“微腐败”。

（二）切实提升政府法治水平。坚持依法行政，严格执行市人大及其常委会决议决定，自觉接受人大法律监督、工作监督和政协民主监督，高质量办好建议提案，主动接受社会监督。抓好重点领域行政立法，持续推进行政决策合法性审查规范化、标准化建设，健全政府法律顾问工作机制。全面推行行政执法“三项制度”改革，推广应用综合行政执法智慧服务平台。加强和改进行政应诉工作。加强宪法和法治宣传教育，严格执行政府规章、规范性文件备案制度。加强依法统计，切实做好第四次全国经济普查。深化政务公开，促进政府数据资源向社会开放。

（三）建设务实高效政府。坚持求真务实、真抓实干，力戒形式主义、官僚主义。健全抓落实工作的制度和机制。认真落实机构改革方案。加强公职人员培训教育，提升专业化水平和履职能力。强化调查研究，提高政策制定和执行水平，突出差异化和协调性，不搞“一刀切”。学习借鉴国内外先进城市经验，发挥好城市智慧治理中心的作用，加强政府核心智库建设，推动政府决策更科学、服务更高效！

各位代表！思想引领时代，核心领航事业。让我们更加紧密团结在以习近平同志为核心的党中央周围，以习近平新时代中国特色社会主义思想为指导，深入贯彻落实习近平总书记对四川及成都工作系列重要指示精神，在党中央国务院、省委省政府和市委坚强领导下，不忘初心、奋发有为，大力推动高质量发展，持续增进民生福祉，加快建设全面体现新发展理念的国家中心城市，以优异成绩迎接中华人民共和国成立70周年！
